# Adverse perinatal outcomes indicative of RhD-mediated hemolytic disease of the fetus and newborn in Eastern Ethiopia: evidence of maternal health inequity in a multicenter cohort study

**DOI:** 10.1016/j.xagr.2026.100625

**Published:** 2026-03-18

**Authors:** Assefa Desalew, C. Ellen van der Schoot, Rafiki N. Mjema, Thomas van den Akker, Tienke Vermeiden, Henk Schonewille, Jeremia J. Pyuza, Tadesse Gure, E.J.T. (Joanne) Verweij, Abera Kenay Tura

**Affiliations:** 1 Department of Pediatric and Child Health Nursing, School of Nursing, College of Health and Medical Sciences (Desalew, Tura), Haramaya University, Harar, Ethiopia; 2 Department of Obstetrics and Gynecology (Desalew, Mjema, van den Akker, Vermeiden, Verweij), Leiden University Medical Centre, Leiden University, the Netherlands; 3 Department of Experimental Immunohematology, Sanquin Research and Landsteiner Laboratory, Academic Medical Center (van der Schoot, Schonewille), University of Amsterdam, Amsterdam, the Netherlands; 4 Department of Obstetrics and Gynecology (Mjema), Kilimanjaro Christian Medical Centre, Moshi, Tanzania; 5 Department of Pathology (Pyuza), Kilimanjaro Christian Medical Centre, Moshi, Tanzania; 6 Department of Obstetrics and Gynecology, College of Health and Medical Sciences (Gure), Haramaya University, Harar, Ethiopia; 7 Department of International Public Health (Tura), Liverpool School of Tropical Medicine, Liverpool, UK

**Keywords:** Ethiopia, Hemolytic disease of the fetus and newborn, Health inequity, Low and middle-income countries, Risk pregnancy, Rh disease, RhD blood group

## Abstract

**BACKGROUND:**

Although hemolytic disease of the fetus and newborn (HDFN) has become rare in high-income countries, it remains a significant cause of perinatal death in low-and middle-income countries. Nonetheless, true epidemiological data in Africa are lacking.

**OBJECTIVE:**

To obtain insight into the prevalence of anti–Rhesus D-mediated HDFN in Ethiopia, pregnancy outcomes were compared between RhD-negative and RhD-positive women.

**STUDY DESIGN:**

A multicenter facility-based retrospective cohort study was performed on 6796 women who gave birth (≥28 weeks of gestation) in 13 Ethiopian Obstetric Surveillance System hospitals from January to March 2024. Data were retrospectively collected from maternal and neonatal medical records from antenatal care to birth and neonatal registry. The composite adverse perinatal outcomes (APO) included stillbirth, neonatal loss, or Neonatal Intensive Care Unit admission. The likelihood of HDFN was based on clinical review by 3 experts blinded to the maternal blood group.

**RESULTS:**

In 6141 of 6796 women with known RhD status, 327 (5.3%) were RhD-negative. APO was seen in 13.3% women, and occurred twice as often in RhD-negative, as compared to RhD-positive women (aOR=2.3; 95% CI: 1.7–2.9). Clinical signs were highly suggestive of HDFN in 5.8% of RhD-negative women, as compared to 0.2% in RhD-positive women (*P*<.0001).

**CONCLUSION:**

Our study identifies that RhD-negative women experience a two-fold increased odds of APO, most likely due to HDFN. This highlights the need for strategies to address these maternal and child health inequities, including using anti-RhD immunoprophylaxis to prevent HDFN and screening for blood group antibodies to identify pregnancies at risk.

**AJOG Global Reports at a Glance**

**Why was this study conducted?**

HDFN is estimated to contribute to over 160,000 perinatal deaths annually. But these estimates rely on Western data. Thus, true data on the burden of HDFN in Africa, including Ethiopia, remain lacking.

**Key findings?**

Adverse pregnancy outcomes are two-fold higher in RhD-negative women as compared to RhD-positive women.

Approximately 5.8% of all RhD-negative women (and 8.3% of RhD-negative multigravida) exhibited clinically significant signs highly suggestive of HDFN, as compared to 0.2% in RhD-positive women.

**What does this add to what is known?**

The study reveals a previously unrecognized inequity in maternal and newborn health: RhD-negative women face twice the odds of adverse perinatal outcomes, likely due to HDFN.

This highlights the need for global strategies such as anti-RhD immunoprophylaxis and blood-group antibody screening to prevent and identify at-risk pregnancies.

## Introduction

RhD is an antigenic protein on the surface of red blood cells (RBCs). Individuals lacking this blood group antigen are termed RhD-negative.^1^ RhD alloimmunization may occur when an RhD-negative pregnant woman is exposed to RhD-positive fetal red blood cells, most commonly during delivery, but also at any time during pregnancy (eg, bleeding, trauma, or invasive procedures). She may then develop an immune response, leading to the production of anti-RhD antibodies. These antibodies can cross the placenta during subsequent pregnancies, causing “Rh disease,” a subtype of hemolytic disease of the fetus and newborn (HDFN).^2^, ^3^, ^4^, ^5^ In severe HDFN, fetal anemia induces fetal hydrops, stillbirth, and perinatal death.^3^^,^^6^^,^^7^ If a newborn survives, persistent hemolysis may cause neonatal anemia, jaundice, or even kernicterus, resulting in neurodevelopmental impairment or death.^7^^,^^8^

Since the introduction of anti-RhD immunoprophylaxis in the 1960s, HDFN, once the major cause of perinatal deaths, has been almost eradicated in high-income countries.^2^ However, there are significant differences in the use of anti-RhD immunoprophylaxis between low- and middle-income countries (LMICs) and high-income countries.^6^ It is estimated that half of all women worldwide requiring postnatal anti-RhD immunoprophylaxis do not receive it, because of substantial barriers to access. This gap is exacerbated by the lack of reliable data on the burden of this disease in LMICs. Historically, HDFN prevalence has been assumed to be low in sub-Saharan Africa, and thus it is not prioritized.^9^^,^^10^ Although anti-RhD immunoprophylaxis is included on the World Health Organization (WHO) essential medicines list, it is limited available in most LMICs.^2^^,^^6^ Additionally, in many low-resource settings, only monoclonal anti-D is available, despite limited evidence supporting its clinical effectiveness.^11^ HDFN is estimated to contribute to over 160,000 perinatal deaths and 100,000 cases of disability annually. However, these estimates are based on extrapolations using historical data from high-income countries.^12^ Reliable “real-world” data are missing, particularly from Africa.^8^^,^^12^ Therefore, it is unknown whether the risk of eliciting a pathogenic immune response to RhD and the corresponding risk of severe HDFN are similar in Africans and Europeans. As described recently, this presumably high burden of a preventable disease, caused by a global inequity in obtaining effective prophylaxis, is unacceptable.^2^^,^^9^^,^^11^ Therefore, the African Initiative for Rh-Disease Eradication (AFRICARhE) project was launched in Ethiopia, Tanzania, and Malawi. AFRICARhE aims to enhance knowledge of HDFN and identify sustainable ways to prevent HDFN and improve care.

Since 2022, the national antenatal care guideline of the Ethiopian Ministry of Health has recommended blood group and RhD typing for all pregnant women and newborns.^13^ However, implementing these recommendations has been inconsistent. Antibody testing is rarely performed, and only in highly selected populations.^14^^,^^15^ Consequently, little is known about the prevalence of blood group alloimmunization and anti-RhD-mediated HDFN in Ethiopia, highlighting a clear knowledge gap in the Ethiopian and broader African context. Robust data on the true disease burden are essential to inform and motivate public health policy change. Therefore, this study aims to provide information regarding HDFN prevalence in Ethiopia. As a proxy for HDFN, we studied the proportion of RhD-negative women who had an adverse perinatal outcomes (APO), as compared to RhD-positive women.

## Materials and methods

### Study setting, design, and population

This retrospective cohort study utilized the Ethiopian Obstetric Surveillance System (EthOSS) network in eastern Ethiopia.^16^ This multicenter surveillance system network enables monitoring of rare and/or severe pregnancy-related conditions. The network includes 13 facilities: 2 referral hospitals, 8 general hospitals, and 3 primary hospitals. These facilities are in the Harari Region, Dire Dawa City Administration, and 2 zones in the eastern Oromia Region (East and West Hararghe). Details of the EthOSS methodology were previously described.^16^ For the present study, data collection tools were adapted to include HDFN-related variables. Data were collected for all births at >28 weeks of gestation occurring in these facilities between January 1 and March 31, 2024. There were no exclusions. The study's ethical approval was obtained from the Institutional Health Research Ethics Review Committee of the College of Health and Medical Sciences, Haramaya University (Ref no: IHRERC/203/2023) (Supplemental Figure 1). As the study involves a retrospective review of records, individual informed consent of the patients was not required. The confidentiality of patients during the medical record review was protected by obtaining permission from the head of the facilities and using only anonymized data with all identifiable information removed.

We followed the Strengthening the Reporting of Observational Studies in Epidemiology (STROBE) Guidelines to report the findings (Supplemental file 1).

### Data sources

Data were retrospectively collected from maternal and neonatal medical records starting from antenatal care to birth and neonatal registry, including laboratory registers. Trained EthOSS research assistants extracted data using REDCap software in April and May 2024. The data collection questionnaire contained several characteristics. These included demographic characteristics, pregnancy-related characteristics for the index and previous pregnancies, perinatal outcomes, and laboratory parameters. The laboratory parameters covered the ABO blood group, RhD type, and anti-RhD-antibodies. The group in which the RhD status was not documented in the medical record was separately studied and compared to the RhD-negative and RhD-positive groups to investigate any potential bias in the results. RBCs, ABO, and RhD blood types were determined using a slide method with anti-A, anti-B, and anti-RhD monoclonal antibodies (Tulip Diagnostics (P) Ltd, Gitanjali, Tulip Block, India). Screening for anti-RhD antibodies is not available in eastern Ethiopia and is only performed in the capital (Addis Ababa) and some private laboratories.

### Outcomes

In the absence of anti-RhD antibody screening, any APO and clinical signs of HDFN were used as a proxy to identify clinically relevant blood group alloimmunization. The composite APO of the index pregnancy was defined as one or more of the following: stillbirth (ie, defined by the World Health Organization as a fetal death >28 weeks of pregnancy, but before or during birth), neonatal death (mortality after birth until 28 days postpartum), clinical signs of HDFN, or admission to the Neonatal Intensive Care Unit (NICU).^17^

Clinical signs suggesting HDFN were defined as: intrauterine transfusion (IUT), stillbirth, hydrops, severe anemia (Hb<7 g/dL), hyperbilirubinemia, and/or icterus requiring phototherapy. The standardized birth registry in Ethiopia includes data points starting from a gestational age of 28 weeks and 0 days.^16^ Pregnancy loss before 28 weeks and 0 days is not recorded as a childbirth; Instead, pregnancy losses before 28 weeks are summarized as "abortion." This category includes miscarriages, late stillbirths, extremely premature births, and induced pregnancy termination.

### Criteria-based clinical score for hemolytic disease of the fetus and newborn

The primary investigator (AD) selected cases from the dataset with a clinical course suggestive of possible APO. Then, the actual probability of HDFN was scored clinically by 2 fetal-maternal medicine specialists (EJTV and TG) and 1 immunohematologist (EvdS). The experts examined the medical records of these women and their newborns. They were all blinded to the mother’s ABO and RhD blood group status. They evaluated the obstetric history (eg, number of abortions, stillbirths, and neonatal losses; number of live births; outcome of previous pregnancies) and the presence of other adverse outcomes (eg, (pre)eclampsia, congenital anomalies, placental abruption, neonatal sepsis). In particular, the combination of multiple fetal and/or neonatal losses with increasing severity in subsequent pregnancies made the diagnosis more suggestive of HDFN. Clinical assessors independently scored HDFN on a 7-point scale, with 1 meaning "unlikely to be HDFN," due to clear other reasons for APO and/or no signs of anemia or jaundice, to 7 being "highly likely HDFN." Cases without sufficient information were scored as 3. All cases that were scored as 6 (might be HDFN) or 7 (high likelihood HDFN) by at least 1 expert were discussed. Cases in which scores were 3 or more points apart were also discussed between the experts. Scores were modified based on this discussion. For the final scores of 6 or 7, the 3 experts did not deviate more than 1 point. Median scores were used for the final analysis before unblinding the maternal blood group status (Supplemental file 2).

### Statistical analysis

The data were analyzed using Statistical Package for the Social Sciences (SPSS) v29. Descriptive statistics were performed and reported using frequencies, percentages, means, and standard deviations. A Chi-square (χ^2^) test was performed to compare the differences between RhD-positive and RhD-negative women. The associations between potential risk factors and APO were assessed using a binary logistic regression. The following variables were chosen for the model: RhD type, ABO blood group, antenatal care, maternal age, referral status, residence, history of stillbirth, and history of abortion. These choices were based on prior literature, biological plausibility, and their potential as confounding factors influencing APO and blood group alloimmunization. The association was described using an aOR with a 95% CI. Multi-collinearity was checked using a variance inflation factor (>10) and standard error (>2). Goodness-of-fit was checked using the Hosmer–Lemeshow test (>0.05). Statistical significance was set at *P*<.05 in the multivariable logistic regression.

### Patient and public involvement

Patients and the public were not involved in study design, data collection, data analysis, data interpretation, writing, or dissemination plans of this research.

## Results

### Maternal characteristics

A total of 6796 maternal and neonatal medical records were included in the study. The mean maternal age was 25.4 (±5.4) years, with over 80% of women aged between 18 and 35 years. Half the women were urban residents (3417, 50.3%), and 2146 (31.6%) were primigravida. A total of 1426 (21.0%) had been referred from lower-level health facilities, and of these, 1163 (81.6%) had been referred from a health center (Table 1).

**Table 1:** Maternal characteristics by RhD-status among women who gave birth in public hospitals in eastern Ethiopia between January 1 and March 31, 2024

Variables	Categories	RhD type			
		Negative (n=327)	Positive (n=5814)	Unknown (n=655)	Total (n=6796)
Maternal age in years	<18	33 (10.1)	513 (8.8)	60 (9.2)	606 (8.9)
	18–35	262 (80.1)	4831 (83.1)	527 (80.5)	5620 (82.7)
	>35	32 (9.8)	470 (8.1)	68 (10.4)	570 (8.4)
Residence	Urban	174 (53.2)	2961 (50.9)	282 (43.1)	3417 (50.3)
	Rural	153 (46.8)	2853 (49.1)	373 (56.9)	3379 (49.7)
Referred	No	262 (80.1)	4595 (79.0)	513 (78.3)	5370 (79.0)
	Yes	65 (19.9)	1219 (21.0)	142 (21.7)	1426 (21.0)
Referred from	Health center	49 (75.4)	990 (81.2)	124 (87.3)	1163 (81.6)
	Primary hospital	2 (3.1)	68 (5.6)	5 (3.5)	75 (5.3)
	General hospital	6 (9.2)	54 (4.4)	9 (6.3)	69 (4.8)
	Referral hospital	1 (1.5)	10 (0.9)	0 (0)	11 (0.7)
	Private facility	7 (10.6)	97 (8.0)	4 (2.8)	108 (7.6)
Gravidity	Primigravida	97 (29.7)	1841 (31.7)	208 (31.8)	2146 (31.6)
	Multigravida	171 (52.3)	2802 (48.2)	306 (46.7)	3279 (48.2)
	Grand multigravida	59 (18.0)	1171 (20.1)	141 (21.5)	1371 (20.2)
Parity	Primiparous (1st birth)	108 (33.0)	2032 (35.0)	225 (34.4)	2365 (34.8)
	Multiparous (1–4 births)	181 (55.4)	3126(53.8)	343 (52.4)	3650 (53.7)
	Grand multiparous (≥5 births)	38 (11.6)	656 (11.3)	87 (13.3)	781 (11.5)

Data are presented as numbers (%).

In 655 of the 6796 (9.6%) women, blood group status was not screened or recorded. Among the 6141 women with known blood groups, 327 (5.3%) (95% CI: 4.8–6.0) were RhD-negative. The ABO blood group distribution was as follows: 291 (4.8%) AB, 1315 (21.4%) B, 1853 (30.2%) A, and 2682 (43.7%) O. There were no statistical differences between women who were RhD-negative, RhD-positive, or of unknown blood group for many of the maternal characteristics. The only exception was for residence: women of unknown blood group were more likely to live in a rural setting (Table 1). The ABO blood group distribution was similar between RhD-positive and RhD-negative women (Supplemental Figure 2).

### History of previous pregnancies and outcomes

Of the 4650 women with a history of multiple pregnancies, 924 (19.9%) had a history of "abortion." There were no differences between the proportions of RhD-positive and RhD-negative women with a history of abortion. Of the 48 RhD-negative women with abortion, 4 had a history of ≥3 consecutive abortions (8.3%), as compared to 30 (3.7%) among RhD-positive women.

Two hundred sixty-eight women (5.8%) had a history of stillbirth, and 157 (3.4%) had a history of neonatal loss. Having a history of stillbirth was more frequent among RhD-negative women: 25 (10.9%) compared to 228 (5.7%) among RhD-positive women (OR=2.0; 95% CI: 1.3–3.1; *P*<.0001). No statistical difference was observed in the percentages of RhD-negative and RhD-positive women with a history of neonatal death (Table 2). The group with unknown RhD type was comparable with the RhD positive group for all variables.

**Table 2:** Outcomes of previous pregnancies among women who gave birth in public hospitals in eastern Ethiopia, 2024 (n=4650)

Variables	Categories	RhD type			
		Negative (n=230)	Positive (n=3973)	Unknown (n=447)	Total (n=4650)
History of abortion (=pregnancy loss <28 weeks)	Total	48 (20.9)	802 (20.2)	74 (16.6)	924 (19.9)
Number of abortions (n=924)	One	33 (68.8)	582 (72.6)	57 (77.0)	672 (72.7)
	Two	11 (22.9)	157 (19.6)	12 (16.2)	180 (19.5)
	Three and above	4 (8.3)	63 (7.9)	5 (6.8)	72 (7.8)
Three or more consecutive abortions	Total	4 (8.3)	30 (3.7)	2 (2.7)	36 (3.9)
Onset of abortion (n=924)	Induced	0 (0.0)	29 (3.6)	2 (2.7)	31 (3.4)
	Spontaneous	46 (95.8)	724 (90.3)	68 (91.9)	838 (90.7)
	Not recorded	2 (4.2)	49 (6.1)	4 (5.4)	55 (5.9)
History of stillbirths*	Total	25 (10.9) *	228 (5.7) *	15 (3.4)	268 (5.8)
Number of stillbirths (n=268)	One	17 (68.0)	163 (71.5)	12 (80.0)	192 (71.6)
	Two	6 (24.0)	51 (22.4)	2 (13.3)	59 (22.0)
	Three and above	2 (8.0)	14 (6.1)	1 (6.7)	17 (6.3)
History of neonatal death	Total	10 (4.3)	134 (3.4)	13 (2.9)	157 (3.4)
Number of neonatal deaths (n=157)	One	7 (70.0)	105 (78.4)	9 (69.2)	121 (77.1)
	Two	1 (10.0)	21 (15.7)	3 (23.1)	25 (15.9)
	Three and above	2 (20.0)	8 (6.0)	1 (7.7)	11 (7.0)

Data are presented as numbers (%).

⁎ There is a significant difference between RhD-positive and RhD-negative pregnant women, *P*=.0015.

### Anti-RhD immunoprophylaxis and alloantibody status

Of 327 RhD-negative women, 23 (7.3%) were screened for alloantibodies. Of those screened, 5 (21.7%) had anti-RhD-alloantibodies. None of these were in the APO group and were therefore not classified as possible HDFN cases. Twenty-four (7.3%) of the RhD-negative pregnant women received anti-RhD immunoprophylaxis during the index pregnancy: 1 received both antenatal and postnatal immunoprophylaxis, 16 received only postnatal anti-RhD immunoprophylaxis, and 7 received only antenatal anti-RhD immunoprophylaxis.

### Neonatal and maternal outcome of present pregnancy

There were no statistically significant differences between RhD-positive, RhD-negative women, and women with unknown Rh status concerning pregnancy-related complications (Supplemental Table 2). Upon discharge, 18 maternal deaths had occurred (2 in RhD-negative women). This corresponds to a hospital-based maternal mortality rate of 264 per 100,000 live births. There were no differences between the mean gestational age at birth or neonatal birth weights between RhD-negative and RhD-positive women.

Overall, 904 of the 6796 women had a composite APO (13.3%; 95% CI: 12.5–14.1). This included: 371 stillbirths (5.5%) and 533 NICU admissions (7.8%). Of the NICU admissions, 90 (16.9%) were for neonatal anemia, 31 (5.8%) for severe jaundice, and 33 (6.2%) neonates died.

In the binary logistic regression analysis, APO was independently associated with the RhD-negative blood group (aOR=2.3; 95% CI: 1.7–2.9), a history of stillbirth (aOR=2.0; 95% CI: 1.5–2.7) or pregnancy loss (aOR=1.2; 95% CI: 1.0–1.5), having been referred (aOR=1.5; 95% CI: 1.3–1.8), urban residence (aOR=0.8; (95% CI: 0.7–0.9), and receiving antenatal care (aOR=0.8; 95% CI: 0.7–0.9) (Table 3). Sensitivity analyses were conducted to see the effect of primigravid women (∼31%) and women with missing blood group information (<10%) on the final model. The sensitivity analyses demonstrated that the association between RhD-negative status and APO remained consistent (aOR 2.25–2.40). Minor changes were observed for antenatal care (aOR 1.41 → 1.27), and history of stillbirth (aOR 2.08 → 1.98), but all estimates retained direction and significance, demonstrating robustness (Supplemental file 3).

**Table 3:** Factors associated with adverse perinatal outcomes among women who gave birth in public hospitals in eastern Ethiopia, 2024 (n=6796)

Variables	Categories	APO		cOR (95% CI)	aOR (95% CI)	*P*-value
		Yes	No			(aOR)
RhD type	Negative	81	246	2.3 (1.8–3.0)	2.3 (1.7–2.9)	<.0001
	Unknown	90	565	1.1 (0.9–1.4)	1.1 (0.8–1.4)	.4729
	Positive	733	5081	1.0	1.0	
ABO blood group	A	236	1617	1.0	1.0	
	B	178	1137	1.1 (0.9–1.3)	1.1 (0.9–1.3)	.5297
	AB	44	247	1.2 (0.9–1.7)	1.3 (0.9–1.8)	.2182
	O	356	2326	1.0 (0.9–1.3)	1.0 (0.9–1.2)	.6618
	Unknown	90	565	1.1 (0.8–1.4)	1.1 (0.8–1.4)	.7530
Antenatal care	Yes	408	3057	1.0	1.0	
	No	496	2835	1.3 (1.1–1.5)	1.3 (1.1 – 1.5)	.0013
Maternal age (y)	Less than 18	87	519	1.1 (0.9–1.4)	1.1 (0.9–1.4)	.5151
	Greater than 35	84	486	1.2 (0.9–1.5)	1.0 (0.8–1.3)	.8491
	18–35	733	4887	1.0	1.0	
Referred	Yes	257	1169	1.6 (1.4–1.9)	1.5 (1.3–1.8)	<.0001
	No	647	4723	1.0	1.0	
Residence	Urban	385	3032	0.7 (0.6–0.8)	0.8 (0.7–0.9)	<.0001
	Rural	519	2860	1.0	1.0	
History of stillbirth	Yes	65	203	2.3 (1.7–3.1)	2.0 (1.5–2.7)	<.0001
(n=4650)	No	529	3853	1.0	1.0	
History of abortion	Yes	139	785	1.2 (1.0–1.6)	1.2 (1.0–1.5)	.0410
(n=4650)	No	455	3271	1.0	1.0	

APO, Adverse Perinatal Outcomes; cOR, crude odds ratio; aOR, adjusted odds ratio.

Hosmer and Lemeshow Test (*P*=.8018); Multi-collinearity test: Tolerance values for all variables was above 0.1, and most are above 0.9; variance inflation factor (VIF) values are all close to 1, far below the common thresholds of 5 or 10.

The outcome of pregnancies of RhD-negative women was compared with that of those who were RhD-positive. This was used as a reflection of the possible consequences of alloimmunization. There was a significant difference in NICU admission (OR=2.5; 95% CI: 1.8–3.4; *P*<.0001). Among the NICU-admitted babies, the prevalence of neonatal anemia (OR=2.3; 95% CI: 1.2–4.2; *P*=.0100) and severe jaundice (OR=9.4; 95% CI: 4.3–20.3; *P*<.0001) was higher in babies of RhD-negative women compared to RhD-positive women. Again, the outcome of the pregnancy of women with an unknown blood group was comparable with that of the RhD-positive group. The clinical assessment in women with an APO revealed that HDFN was highly suspected (ie, scores 6 and 7) in 19/81 (23.5 %) of RhD-negative women. This was significantly different from the rate in RhD-positive women, where HDFN was highly suspected in only 15/733 (2.0%) (OR=14.7; 95% CI: 7.1–30.3; *P*<.0001). It was also significantly different from women of unknown blood type, where 2/90 (2.2%) were highly suspected.

In RhD-negative women with babies who were highly suspected to have HDFN, the ABO blood group distribution did not differ from the entire group. However, among RhD-positive women whose infants were highly suspected of having HDFN, blood group O seemed slightly overrepresented: (67% (10/15 in Table 4) compared to 44% (2682/6141 in Table 3) in the total group of women with known ABO blood group; *P*=.072). Crucially, in only 4/19 babies from RhD-negative women, who were suspected to have HDFN, was RhD incompatibility mentioned in the medical chart. In 3 other women, hydrops fetalis (n=2) and kernicterus (n=1) were recorded, but HDFN or Rh incompatibility was not mentioned.

**Table 4:** Distribution of adverse perinatal outcomes and clinical scoring for HDFN among women who gave birth in public hospitals in eastern Ethiopia, 2024.

Variables		RhD type				p-value
		Negative (n=81)	Positive (n=733)	Unknown (n=90)	Total (n=904)	
Adverse perinatal outcome		81 (24.8)	733 (12.6)	90 (13.7)	904 (13.3)	<0.0001
Stillbirth (Index pregnancy)		24 (7.3)	295 (5.1)	52 (7.9)	371 (5.5)	0.0939
NICU admission (n=533)		55 (16.8)	437 (7.5)	41 (6.3)	533 (7.8)	<0.0001
Neonatal anemia*		17 (30.9)	69 (15.8)	4 (9.8)	90 (16.9)	0.0100
Severe jaundice*		15 (27.3)	16 (3.7)	0 (0.0)	31 (5.8)	<0.0001
Neonatal death*		4 (7.2)	27 (6.2)	2 (4.9)	33 (6.2)	0.7826
Hydrops fetalis		2 (0.6)	0	0	2	
**Criteria-based Score for HDFN for 904 cases with APO**						
Unlikely (score 1-2)		48 (59.3)	595 (81.2)	70 (77.8)	713 (78.9)	
Inconclusive (score 3-5)		14 (17.3)	123 (16.8)	18 (20.0)	155 (17.1)	
Highly suspected (score 6-7)		19 (23.5)	15 (2.0)	2 (2.2)	36 (4.0)	<0.0001
Maternal blood group (For the 36 highly suspected HDFN cases)	A	5 (26.3)	2 (13.3)	0 (0.0)	7 (19.4)	
	B	3 (15.8)	2 (13.3)	0 (0.0)	5 (13.9)	
	AB	1 (5.3)	1 (6.7)	0 (0.0)	2 (5.6)	
	O	10 (52.6)	10 (66.7)	0 (0.0)	20 (55.6)	
	Unknown	0 (0.0)	0 (0.0)	2 (100.0)	2 (5.6)	

Data are presented as numbers (%).

NICU, Neonatal Intensive Care Unit; APO, Adverse Perinatal Outcome; HDFN, Hemolytic Disease of the Fetus and Newborn.

⁎ The percentages are among neonates admitted to the NICU; *P*-value refers to the comparison of RhD-negative and RhD-positive.

All 19 RhD-negative women whose babies were scored as highly suspect for HDFN were multigravida. In the total group, however, only 230/327 (70.3%) RhD-negative women were multigravida. This means that at least 19/230 (8.3%) of multigravida women had clinically suspected anti-RhD-mediated HDFN (Table 4), whereas none occurred in RhD-negative women who were primigravida. Additionally, the likely HDFN cases in RhD-positive women occurred only in multigravida.

## Discussion

### Principal findings

This study compared APO between 327 RhD-negative women (5.3%, 95% CI: 4.8–6.0%) and 5814 RhD-positive women. This comparison was used as a proxy to determine the burden of HDFN in Eastern Ethiopia. Multivariable logistic regression analysis showed that RhD-negativity was the strongest risk factor for APO. RhD-negative women have twice the odds of developing APO compared to RhD-positive women; Anti-RhD-mediated HDFN is the most likely explanation for this difference, because there are no other differences between RhD-positive and RhD-negative women that might result in an APO.^18^ Indeed, within the NICU-admitted neonates, the prevalence of severe anemia and jaundice was far higher in babies of RhD-negative women than in those of RhD-positive women. Furthermore, the clinical diagnosis of HDFN was suspected almost 15 times more frequently in RhD-negative than in RhD-positive women. Additionally, access to anti-RhD immunoprophylaxis is inconsistent in LMICs. In our study, only 7.3% of RhD-negative pregnant women were documented as receiving it. Altogether, our data strongly suggest that anti-RhD-mediated HDFN is a relevant and neglected health problem, likely occurring in 5.8% (95% CI: 3.5–8.9) of RhD-negative women. We believe this figure is an underestimation. Primigravida are overrepresented in our cohort, whereas HDFN typically affects multigravida. Moreover, the most severe cases are unlikely to be included in our study, as these women typically lose their children before 28 weeks of gestation. Furthermore, mild cases of HDFN would have been missed in our study, as those cases would not be scored as highly likely to have HDFN by our experts.

### Results in the context of what is known

The frequency of RhD negativity in Eastern Ethiopia (5.3%) is similar to previous studies of pregnant women in Ethiopia (2.1%–12.1%).^14^^,^^19^, ^20^, ^21^ In Africa, neonatal jaundice is often caused by ABO HDFN.^22^ Although anti-A or anti-B are less likely than anti-RhD to cause severe disease.^7^ In our study, ABO HDFN probably accounts for the low percentage of HDFN cases encountered among RhD-positive women. Our data on probable anti-RhD-mediated HDFN agree with a recent Ethiopian study. In that study, 5.2% (17/328) of RhD-negative women had HDFN-related pregnancy complications.^21^ This figure might seem lower than the 16% alloimmunization rate observed in Europeans before the implementation of anti-RhD prophylaxis.^23^ It might be true that the alloimmunization rate is lower in Ethiopia, as this risk depends on genetic factors along with the immune activation status of the women. However, not all women who made anti-RhD antibodies would have been recognized in our study. Cases with mild or no HDFN would be missed. The natural course of Rh disease before implementing anti-RhD immunoprophylaxis showed that anti-RhD antibodies induced severe disease in only 1/3 of the cases. In addition, currently, less than half of the immunized women require intrauterine or exchange transfusions and/or intensive phototherapy. Furthermore, ∼30% of these women give birth to babies without any clinical signs of hemolysis.^21^^,^^24^

### Clinical implications

Given the striking number of APO and deaths seen in this study, addressing HDFN offers a clear opportunity for improvement. HDFN is likely responsible for ∼50% of the APO in RhD-negative women. Its prevention is well understood: 1 postpartum injection of anti-RhD immunoprophylaxis prevents alloimmunization in >90% of cases. However, in this study, only ∼7% of women (ie, 24/327) received anti-RhD immunoprophylaxis, which is lower than those in other LMICs.^12^ Both polyclonal and monoclonal anti-RhD immunoprophylaxis are registered in Ethiopia, but their availability is limited, particularly for the polyclonal anti-RhD. As noted previously, the effectiveness of monoclonal anti-RhD immunoprophylaxis has not been thoroughly studied, and in vitro research indicates it may have no effect.^11^^,^^25^ Additionally, the relatively prohibitive cost, combined with a lack of financial support or insurance, contributes to low utilization among RhD-negative women.

To improve maternal and neonatal health outcomes related to HDFN, national policies should prioritize increasing the availability and affordability of polyclonal anti-RhD immunoprophylaxis. Strategies could include subsidizing costs, incorporating anti-RhD coverage into national health insurance, supporting initiatives for local production of polyclonal anti-RhD immunoprophylaxis, and strengthening supply chain management to ensure equitable access for all eligible RhD-negative women. Addressing these barriers aligns with global health initiatives aimed at reducing preventable cases of HDFN and bridging disparities between LMICs and high-income countries, where polyclonal anti-RhD immunoprophylaxis is widely accessible.^11^^,^^26^ Efforts to produce polyclonal anti-RhD immunoprophylaxis locally in Ethiopia are currently being considered, but this will require time and coordinated policy action.

### Research implications

Guidelines already exist advising blood group typing and anti-RhD immunoprophylaxis to prevent HDFN.^13^ However, health systems should ensure blood typing and HDFN education to create more awareness. Nonetheless, in Ethiopia, there is local awareness as evidenced by a specific descriptor for the disease: “shotelai.” Furthermore, in Addis Ababa, IUTs have been performed for several years.^27^ Finally, a prospective study is planned to determine the alloimmunization rate and the prevalence of anti-RhD-mediated HDFN in a cohort of pregnant women visiting facilities for antenatal care or following pregnancy termination.

### Strengths and limitations

One strength of the current study is that it utilized EthOSS, an already standardized, comprehensive, and multicenter birth data registry. This registry was adapted for HDFN data collection. The detailed data, including access to actual medical records, in the absence of antibody testing, allowed experts to assess the diagnosis of probable HDFN. This assessment appears highly dependable given the significant differences in the HDFN frequency when comparing RhD-negative and RhD-positive women. By analyzing the group of women with unknown RhD status, it was excluded that the missingness of RhD data introduced a bias in the data. Nonetheless, a main limitation is that the findings were based on a retrospective review of routine medical records and the reliance on a clinical proxy outcome rather than immunological confirmation of HDFN. This may result in incomplete information and likely only allow recognition of the most obvious HDFN cases. Moreover, only women who gave birth after 28 weeks of gestation were included. Without antenatal treatment, severe HDFN can result in pregnancy loss before the 28th week, thereby underestimating the burden of HDFN in our study.

## Glossary

**Table:** 

AFRICARhE	African Initiative for Rh-Disease Eradication
anti-RhD	Anti-Rhesus D
aOR	Adjusted odds ratio
APO	Adverse perinatal outcomes
CI	Confidence interval
cOR	Crude odds ratio
EthOSS	Ethiopian Obstetric Surveillance System
HDFN	Hemolytic disease of the fetus and newborn
HICs	High-income countries
IUT	Intrauterine transfusion
LMICs	Low and middle-income countries
NICU	Neonatal Intensive Care Unit
RBCs	Red blood cells
RhD	Rhesus D
SPSS	Statistical Package for the Social Sciences
WHO	World Health Organization

## Conclusion

This study identifies a hitherto barely recognized healthcare inequity that must be addressed urgently. RhD-negative women face a 2-fold increased risk of APO, most likely due to HDFN, which is a preventable condition. In ∼25% of these women, there was a strong indication that the APO was caused by HDFN. We recommend that all women have access to blood group typing and that RhD-negative women be screened for anti-RhD antibodies. Providing RhD-negative women who gave birth to an RhD-positive child with polyclonal anti-RhD immunoprophylaxis will reduce APO due to HDFN, thereby preventing this previously hidden inequity.

## CRediT authorship contribution statement

**Assefa Desalew:** Writing – original draft, Project administration, Methodology, Investigation, Formal analysis, Data curation, Conceptualization. **C. Ellen van der Schoot:** Writing – review & editing, Writing – original draft, Validation, Supervision, Methodology, Investigation, Formal analysis, Conceptualization. **Rafiki N. Mjema:** Writing – review & editing, Validation, Methodology, Formal analysis. **Thomas van den Akker:** Writing – review & editing, Validation, Supervision, Methodology. **Tienke Vermeiden:** Writing – review & editing, Validation, Project administration. **Henk Schonewille:** Writing – review & editing, Validation, Formal analysis. **Jeremia J. Pyuza:** Writing – review & editing, Validation, Investigation, Conceptualization. **Tadesse Gure:** Writing – review & editing, Validation, Supervision, Formal analysis. **E.J.T. (Joanne) Verweij:** Writing – review & editing, Validation, Supervision, Methodology, Investigation, Funding acquisition, Formal analysis, Conceptualization. **Abera Kenay Tura:** Writing – review & editing, Validation, Supervision, Methodology, Investigation, Funding acquisition, Conceptualization.

## Declaration of competing interest

The authors reports no conflict of interest.

## Appendix Supplementary materials

Image, application 1

Image, application 2

Image, application 3

Image, application 4

Image, application 5

Image, application 6

Supplemental Figure S1: ethical approval letter Image, application 7
