# Supplementary material for: Adverse perinatal outcomes indicative of RhD-mediated hemolytic disease of the fetus and newborn in Eastern Ethiopia: evidence of maternal health inequity in a multicenter cohort study
Source: AJOG Glob Rep. 2026 Mar 18;6(2):100625. doi: 10.1016/j.xagr.2026.100625 (PMC13101771; doi:10.1016/j.xagr.2026.100625)
Supplement: Supplementary file 1 [file mmc1.pdf]

Ref. No. IHRERC /203/2023

November 08/2023

To: Chief Executive Director, College of Health and Medical Sciences (COHMS), Haramaya University, Harar Campus, Ethiopia.

From: Institutional Health Research Ethics Review Committee (IHRERC), College of Health and Medical Sciences, Harar Campus, Ethiopia.

ነጋ ባሬሳ
Nega Baresa

Chair-person of the IHRERC

Haramaya University College of Health and Medical Sciences Institutional Health Research Ethics Review Committee (IHRERC) Harar Ethiopia

Subject: Ethical approval of a research proposal by Dr. Abera Kenay Tura (PhD) et al, Academic Staffs of the COHMS – Haramaya University and Collaborators from Others.

It is known that various research proposals are passing through the IHRERC for ethical review. To this effect **Dr. Abera Kenay Tura (PhD)[1], as Principal Investigator (PI) and as Co- Investigators Dr. Tadesse Gure (MD)[1], Prof. Thomas van den Akker (MD, PhD)[2], Dr. Joanne Verweij (MD, PhD)[2], Prof. Dr. Ellen van der[2], Dr. Bariki Lawerence[3], Dr. Rafiki Nickson Mjema[3], Dr. Jeremia J. Pyuza[3], Priscus Jhon Mapendo[3], Dr. Jeremiah John Hhera[3], Drs Derek P de Winter[2], Drs. Renske van 't Over[2], Assefa Desalew[1], and Kabtamu Gemechu[1]** have submitted a research proposal entitled "**Burden of Hemolytic Disease of the Fetus and Newborn and Introduce context-specific interventions in selected Hospitals in Eastern Ethiopia"** through your office to the IHRERC for ethical review and approval (with a letter written by the PI on September 02/2023 and directed by your esteemed office to office of the IHRERC on 22/10/2016 E.C). The IHRERC has scrutinized the proposal for ethical issues and made the investigators to correct and incorporate essential elements. The investigators, therefore, have incorporated all elements as enquired by the committee. The IHRERC has, thus, approved for implementation the 89 pages proposal unanimously through full consensus of the currently existing eight members on its regular meeting convened on November 07/2023. The IHRERC congratulates the investigators for the concerted efforts they made to fulfill the recommendations of the Committee.

Finally the IHRERC requests your Office, to inform officially the investigators to commence their data collection process by contacting for permission of the concerned authorities in the respected study area/ setting; and **strictly following the international precautions to prevent disease transmissions.** However, since the IHRERC is bestowed to make follow-up of the research process, the investigators are informed with a copy of this letter to report any changes in the research procedure and submit an activity progress report to the IHRERC **every three months.** A copy of the final report is also expected.

At the back of this letter please find the approval format of the IHRERC. One signed and stamped copy of the approved proposal document is also attached.

With Regards

CC:

- **Dr. Abera Kenay Tura (PhD)[1], Dr. Tadesse Gure (MD)[1], Prof. Thomas van den Akker (MD, PhD)[2], Dr. Joanne Verweij (MD, PhD)[2], Prof. Dr. Ellen van der[2], Dr. Bariki Lawerence[3], Dr. Rafiki Nickson Mjema[3], Dr. Jeremia J. Pyuza[3], Priscus Jhon Mapendo[3], Dr. Jeremiah John Hhera[3], Drs Derek P de Winter[2], Drs. Renske van 't Over[2], Assefa Desalew[1], and Kabtamu Gemechu[1]** (including one copy of the approved proposal)

Affilations: [1] College of health and Medical Sciences, Haramaya University [2] Leiden University Medical Center [3]Kilmanjaro Christian Medical Center

Haramaya University, College of Health and Medical Sciences
Institutional Health Research Ethics Review Committee (IHRERC)
Address: Tel.0254662011 P.O.Box 235 Fax 0256668081, Harar-Ethiopia
e-mail neggalemash@gmail.com

Institutional Health Research Ethics Review Approval Form

Name of the institution: College of Health and Medical Sciences, Haramaya University, Harar Campus

Name of PI: Dr. Abera Kenay Tura (PhD) - Tel +251912048026, Email: daberaf@gmail.com P.O.Box 235, Harar, Ethiopia.

Title of the proposal/project: Burden of Hemolytic Disease of the Fetus and Newborn and Introduce context-specific interventions in selected Hospitals in Eastern Ethiopia.

To: Office of the Chief Executive Director, College of Health and Medical Sciences, Haramaya University, Harar Campus, Ethiopia.

The IRERC has reviewed the aforementioned project proposal with special emphasis on the following points:

1. Are all ethical principles considered?

	Yes		No	
1.1 Respect for persons	Yes	√	No	
1.2 Beneficence	Yes	√	No	
1.3 Justice	Yes	√	No	
2. Are the objectives of the study ethically achievable?	Yes	√	No	
3. Is/ Are method(s) ethically sound?	Yes	√	No	

Based on the above mentioned ethical assessment the Institutional Research Ethics Review Committee has

a) **Approved the proposal for**: i. Regional/National Review [ ]
ii. **Implementation** [√]

Expiry date of the approval

**31**	**10**	**2024**
Date	month	year

b) **Conditionally approved** [ ]

c) **Not approved** [ ]

Finally we would like to take this opportunity to request your good office to facilitate his request for the proposed study.

With Best Regards

Chairperson: **Negga Baraki** (Name) — Signature: ነጋ ባራኪ, Nega Baraki — Date: **08/11/2023**

Secretary: Berhe G/Michael (Name) — Signature: for — Date: **08/11/2023**

N.B

For any information, question or clarification don't hesitate to contact the Committee with the addresses at the top.
