# Supplementary material for: Adverse perinatal outcomes indicative of RhD-mediated hemolytic disease of the fetus and newborn in Eastern Ethiopia: evidence of maternal health inequity in a multicenter cohort study
Source: AJOG Glob Rep. 2026 Mar 18;6(2):100625. doi: 10.1016/j.xagr.2026.100625 (PMC13101771; doi:10.1016/j.xagr.2026.100625)
Supplement: Supplementary file 2 [file mmc2.docx]

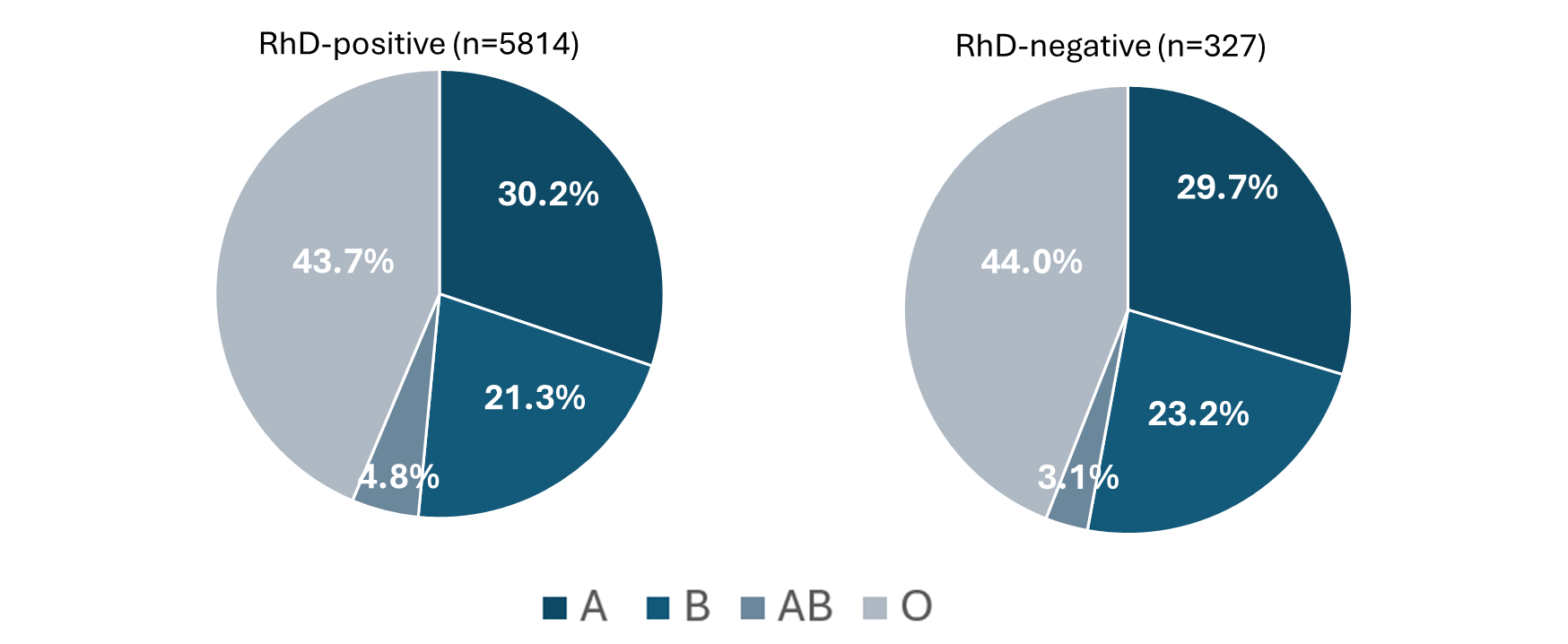


***Figure S2:*** *Distribution of maternal blood group and Rhesus D status among screened pregnancies in public hospitals in eastern Ethiopia, 2024 (n= 6141).*
