# Supplementary material for: Adverse perinatal outcomes indicative of RhD-mediated hemolytic disease of the fetus and newborn in Eastern Ethiopia: evidence of maternal health inequity in a multicenter cohort study
Source: AJOG Glob Rep. 2026 Mar 18;6(2):100625. doi: 10.1016/j.xagr.2026.100625 (PMC13101771; doi:10.1016/j.xagr.2026.100625)
Supplement: Supplementary file 4 [file mmc4.docx]

| Score | The criteria-based score for HDFN is described as follows | Conclusion |
| --- | --- | --- |
| 1 | 1. Cases with stillbirth or neonatal loss in this pregnancy or NICU admission AND 2. No history of stillbirth or neonatal loss AND congenital anomalies or neonatal infection 3. Cases with NICU admission and NO severe jaundice or anemia | Unlikely to have HDFN |
| 2 | 1. Cases with no history of stillbirth or neonatal loss AND NICU admission AND mother with eclampsia 2. Cases with stillbirth or neonatal loss AND abruptio placenta) 3. Cases with NICU AND live discharge of newborn AND placenta previa | Less likely to have HDFN |
| 3 | Cases with no history of stillbirth or neonatal loss AND NICU AND live or unknown discharge of newborn | No positive reason to believe it is HDFN |
| 4 | 1. Cases with no history of stillbirth or neonatal loss AND stillbirth or neonatal loss in this pregnancy (without severe jaundice) 2. Cases with no history of stillbirth or neonatal loss but with severe jaundice in this pregnancy | Fewer reasons to believe it are HDFN |
| 5 | 1) Cases with a history of one stillbirth or neonatal loss AND stillbirth or neonatal loss in this Pregnancy | Considerable reason to believe it is HDFN |
| 6 | 1) Cases with a history of stillbirth or history of neonatal loss AND severe jaundice or anemia or  2) Cases with a history of two or more stillbirths and no stillbirth in the present pregnancy and NICU admission | Might have HDFN |
| 7 | 1) Cases with two or more stillbirths and stillbirth in the present pregnancy or severe anemia or jaundice OR history of Record HDFN | High likely HDFN |
