# Supplementary material for: Adverse perinatal outcomes indicative of RhD-mediated hemolytic disease of the fetus and newborn in Eastern Ethiopia: evidence of maternal health inequity in a multicenter cohort study
Source: AJOG Glob Rep. 2026 Mar 18;6(2):100625. doi: 10.1016/j.xagr.2026.100625 (PMC13101771; doi:10.1016/j.xagr.2026.100625)
Supplement: Supplementary file 5 [file mmc5.docx]

**Model 1: unknown for blood group, and primigravid women were excluded**

| Variables in the Equation | | | | | | | | | |
| --- | --- | --- | --- | --- | --- | --- | --- | --- | --- |
|  | | B | S.E. | Wald | df | Sig. | Exp(B) | 95% C.I.for EXP(B) | |
|  |  |  |  |  |  |  |  | Lower | Upper |
| Step 1^a^ | afri4_referred(1) | .540 | .107 | 25.315 | 1 | .000 | 1.715 | 1.390 | 2.116 |
|  | afri102_residence(1) | -.227 | .097 | 5.498 | 1 | .019 | .797 | .659 | .963 |
|  | ANC follow up(1) | .345 | .095 | 13.102 | 1 | .000 | 1.412 | 1.171 | 1.702 |
|  | maternal age recat |  |  | 1.441 | 2 | .486 |  |  |  |
|  | maternal age recat(1) | -.268 | .303 | .787 | 1 | .375 | .765 | .423 | 1.383 |
|  | maternal age recat(2) | .104 | .138 | .564 | 1 | .453 | 1.109 | .847 | 1.453 |
|  | afri603_maternal_rhd(1) | .875 | .163 | 28.857 | 1 | .000 | 2.398 | 1.743 | 3.299 |
|  | afri207history_stillbirth(1) | .731 | .158 | 21.472 | 1 | .000 | 2.078 | 1.525 | 2.830 |
|  | afri201_history_abortion(1) | .319 | .111 | 8.227 | 1 | .004 | 1.376 | 1.106 | 1.711 |
|  | maternal blood group recat |  |  | .677 | 3 | .879 |  |  |  |
|  | maternal blood group recat(1) | -.038 | .134 | .082 | 1 | .775 | .962 | .740 | 1.252 |
|  | maternal blood group recat(2) | .143 | .222 | .416 | 1 | .519 | 1.154 | .747 | 1.783 |
|  | maternal blood group recat(3) | .020 | .111 | .032 | 1 | .859 | 1.020 | .821 | 1.267 |
|  | Constant | -2.288 | .124 | 339.192 | 1 | .000 | .102 |  |  |
| a. Variable(s) entered on step 1: afri4_referred, afri102_residence, ANC follow up, maternal age recat, afri603_maternal_rhd, afri207history_stillbirth, afri201_history_abortion, maternal blood group recat. | | | | | | | | | |

**Model 2: unknown for blood group and primigravid women included**

| Variables in the Equation | | | | | | | | | |
| --- | --- | --- | --- | --- | --- | --- | --- | --- | --- |
|  | | B | S.E. | Wald | df | Sig. | Exp(B) | 95% C.I.for EXP(B) | |
|  |  |  |  |  |  |  |  | Lower | Upper |
| Step 1^a^ | afri4_referred(1) | .424 | .083 | 26.410 | 1 | .000 | 1.529 | 1.300 | 1.797 |
|  | afri102_residence(1) | -.259 | .075 | 11.798 | 1 | .001 | .772 | .666 | .895 |
|  | ANC follow up(1) | .238 | .074 | 10.221 | 1 | .001 | 1.269 | 1.096 | 1.468 |
|  | maternal age recat |  |  | .439 | 2 | .803 |  |  |  |
|  | maternal age recat(1) | .081 | .124 | .424 | 1 | .515 | 1.084 | .850 | 1.384 |
|  | maternal age recat(2) | .024 | .127 | .036 | 1 | .849 | 1.024 | .799 | 1.314 |
|  | maternal blood group recat |  |  | 1.820 | 4 | .769 |  |  |  |
|  | maternal blood group recat(1) | .068 | .108 | .395 | 1 | .530 | 1.070 | .866 | 1.322 |
|  | maternal blood group recat(2) | .221 | .180 | 1.516 | 1 | .218 | 1.248 | .877 | 1.775 |
|  | maternal blood group recat(3) | .040 | .091 | .191 | 1 | .662 | 1.041 | .871 | 1.244 |
|  | maternal blood group recat(4) | .097 | .135 | .515 | 1 | .473 | 1.102 | .845 | 1.437 |
|  | maternal RhD recat 2 |  |  | 35.767 | 1 | .000 |  |  |  |
|  | maternal RhD recat 2(1) | .813 | .136 | 35.767 | 1 | .000 | 2.254 | 1.727 | 2.941 |
|  | history of stillbirth recat(1) | .683 | .150 | 20.634 | 1 | .000 | 1.981 | 1.475 | 2.660 |
|  | history of aborthion recat(1) | .209 | .102 | 4.175 | 1 | .041 | 1.232 | 1.009 | 1.505 |
|  | Constant | -2.151 | .097 | 487.064 | 1 | .000 | .116 |  |  |
| a. Variable(s) entered on step 1: afri4_referred, afri102_residence, ANC follow-up, maternal age recat, maternal blood group recat, maternal RhD recat 2, history of stillbirth recat, history of abortion recat. | | | | | | | | | |

To assess the robustness of our findings, we conducted sensitivity analyses by comparing two alternative logistic regression models by excluding cases with missing blood group and primigravida women. Effect estimates remained stable across models, supporting the robustness of the findings.

slightly different variable specifications and coding.

| Variable | Model 1 aOR (95% CI) | Model 2 aOR (95% CI) | Change / Interpretation |
| --- | --- | --- | --- |
| RhD-negative | 2.40 (1.74–3.30) | 2.25 (1.73–2.94) | Minimal change; effect remains strong and significant |
| ANC (no follow-up) | 1.41 (1.17–1.70) | 1.27 (1.10–1.47) | Slight decrease; effect remains significant |
| History of stillbirth | 2.08 (1.53–2.83) | 1.98 (1.48–2.66) | Very similar; effect remains significant. |
| Referral | 1.72 (1.39–2.12) | 1.53 (1.30–1.80) | Slight decrease; effect remains significant |
| Residence (urban) | 0.80 (0.66–0.96) | 0.77 (0.67–0.90) | Very similar; effect remains protective. |

The association between RhD-negative status and adverse pregnancy outcomes remained consistent across both models (aOR 2.25–2.40). Minor changes were observed for ANC, referral, and stillbirth, but all effect estimates retained the same direction and statistical significance. The slight improvement in model fit in Model 2 (-2LL decrease, higher Nagelkerke R²) indicates that findings are robust to different model specifications and coding schemes.
