# Supplementary material for: Adverse perinatal outcomes indicative of RhD-mediated hemolytic disease of the fetus and newborn in Eastern Ethiopia: evidence of maternal health inequity in a multicenter cohort study
Source: AJOG Glob Rep. 2026 Mar 18;6(2):100625. doi: 10.1016/j.xagr.2026.100625 (PMC13101771; doi:10.1016/j.xagr.2026.100625)
Supplement: Supplementary file 6 [file mmc6.docx]

*Table S1: List of AFRICARhE consortium members*

| *First, middle, and Last name | Academic  Degrees | Institutions | City and Country |
| --- | --- | --- | --- |
| Enrico Lopriore | MD, Neonatologist | Department of Pediatrics, Willem-Alexander Children's Hospital, Leiden University Medical Center, | Leiden, the Netherlands |
| Derek P de Winter | MD | Department of Pediatrics, Willem-Alexander Children's Hospital, Leiden University Medical Center | Leiden, the Netherlands |
| Renske van 't Oever | MD | Leiden University Medical Centre | Leiden, the Netherlands |
| Sagni Girma Fage | MPH | College of Health and Medical Sciences, Haramaya University | Harar, Ethiopia |
| Habtamu Bekele Ejigu | MSc | College of Health and Medical Sciences, Haramaya University | Harar, Ethiopia |
| Mohammed Yuya Ebro | MPH | College of Health and Medical Sciences, Haramaya University | Harar, Ethiopia |
| Tamirat Getachew | MSc | College of Health and Medical Sciences, Haramaya University | Harar, Ethiopia |
| Addis Eyeberu | MSc | College of Health and Medical Sciences, Haramaya University | Harar, Ethiopia |
| Bikila Balis | MSc | College of Health and Medical Sciences, Haramaya University | Harar, Ethiopia |
| Usmael Jibro | MSc | College of Health and Medical Sciences, Haramaya University | Harar, Ethiopia |
| Megersa Lemi | MSc | College of Health and Medical Sciences, Haramaya University | Harar, Ethiopia |
| Abraham Negash | MSc | College of Health and Medical Sciences, Haramaya University | Harar, Ethiopia |
| Tegenu Balcha | MSc | College of Health and Medical Sciences, Haramaya University | Harar, Ethiopia |
| Obsan Kassa | MPH | College of Health and Medical Sciences, Haramaya University | Harar, Ethiopia |
| Kabtamu Gemechu, | MSc, Hematologist | College of Health and Medical Sciences, Haramaya University | Harar, Ethiopia |
| Tewodros Tesfa | MSc | College of Health and Medical Sciences, Haramaya University | Harar, Ethiopia |
| Konjit Eshetu | MD, Pediatrician | College of Health and Medical Sciences, Haramaya University | Harar, Ethiopia |
| Priscus John Mapendo | Hematologist | Kilimanjaro Christian Medical Center | Moshi, Tanzania |
| Jeremiah John Hhera | MD | Kilimanjaro Christian Medical Center | Moshi, Tanzania |
| Bariki Mchome - | MD, Obstetrician | Kilimanjaro Christian Medical Center | Moshi, Tanzania |
| Patricia Swai | MD, Obstetrician | Kilimanjaro Christian Medical Center | Moshi, Tanzania |
| Blandina T. Mmbaga | MD Paediatrician | Kilimanjaro Christian Medical Center | Moshi, Tanzania |
| Aisa Shayo | MD, Obstetrician | Kilimanjaro Christian Medical Center | Moshi, Tanzania |
| Peter Moons | MD, Pediatrician | Kamuzu University | Blantyre, Malawi |
| Kondwani Kawaza | MD, Pediatrician | Kamuzu University | Blantyre, Malawi |
| Luis Gadama | MD, Obstetrician | Queen Elizabeth Hospital | Blantyre, Malawi |
