# Supplementary material for: Adverse perinatal outcomes indicative of RhD-mediated hemolytic disease of the fetus and newborn in Eastern Ethiopia: evidence of maternal health inequity in a multicenter cohort study
Source: AJOG Glob Rep. 2026 Mar 18;6(2):100625. doi: 10.1016/j.xagr.2026.100625 (PMC13101771; doi:10.1016/j.xagr.2026.100625)
Supplement: Supplementary file 7 — Supplemental Figure S1: ethical approval letter [file mmc7.docx]

*Table S2: Obstetric characteristics and pregnancy outcomes among women who gave birth in public hospitals in eastern Ethiopia, 2024 (n=6796).*

| Variables | Categories | RhD type | | |  |
| --- | --- | --- | --- | --- | --- |
|  |  | Negative (n=327) | Positive  (n=5814) | Unknown (n=655) | Total  (n=6796) |
| Antenatal care | Yes | 162 (49.5) | 3076 (52.9) | 227 (34.7) | 3465 (51.0) |
|  | No | 165 (50.5) | 2738 (47.1) | 428 (65.3) | 3331 (49.0) |
| Number of antenatal | One | 41 (25.3) | 811 (26.4) | 70 (30.8) | 922 (26.6) |
| care received | Two | 26 (16.0) | 504 (16.4) | 40 (17.6) | 570 (16.5) |
|  | Three | 22 (13.6) | 429 (13.9) | 21 (9.3) | 472 (13.6) |
|  | Four and above | 45 (27.8) | 762 (24.8) | 41 (18.1) | 848 (24.5) |
|  | Not recorded | 28 (17.3) | 570 (18.5) | 55 (24.2) | 653 (18.8) |
| Type of pregnancy (n=6772) | Singleton | 315 (96.6) | 5606 (96.7) | 621 (96.0) | 6542 (96.6) |
|  | Multiple | 11 (3.4) | 193 (3.3) | 26 (4.0) | 230 (3.4) |
| Gestational age | Preterm | 71 (21.7) | 1305 (22.4) | 151 (23.1) | 1527 (22.5) |
|  | Term | 251 (76.8) | 4429 (76.2) | 504 (76.9) | 5184 (76.3) |
|  | Post-term | 5 (1.5) | 80 (1.4) | 0 (0.0) | 85 (1.3) |
| Mode of birth | Spontaneous VD* | 240 (73.4) | 4241 (72.9) | 553 (84.4) | 5034 (74.1) |
|  | Instrumental | 1 (0.3) | 64 (1.1) | 10 (1.5) | 75 (1.1) |
|  | Cesarean section | 86 (26.3) | 1509 (26.0) | 92 (14.0) | 1687 (24.8) |
| Obstetric | PROM* | 22 (6.7) | 556 (9.6) | 50 (7.6) | 628 (9.2) |
| complications | PIH* | 16 (4.9) | 413 (7.1) | 43 (6.6) | 472 (6.9) |
| (n=1701) | APH* | 18 (5.5) | 222 (3.8) | 6 (0.9) | 246 (3.6) |
|  | Oligohydramnios | 13 (4.0) | 204 (3.5) | 13 (2.0) | 230 (3.4) |
|  | Macrosomia | 2 (0.6) | 63 (1.1) | 6 (0.9) | 71 (1.0) |
|  | PPH* | 6 (1.8) | 47 (0.8) | 1 (0.2) | 54 (0.8) |
| Maternal hemoglobin | <7 | 11 (3.7) | 165 (3.1) | 5 (2.1) | 181 (3.1) |
| at admission (mg/dl) | 7-9 | 28 (9.3) | 453 (8.4) | 17 (7.0) | 498 (84) |
| (n=5916) | 9-11 | 80 (26.7) | 1343 (25.0) | 70 (28.9) | 1493 (25.2) |
|  | ≥11 | 181 (60.3) | 3413 (63.5) | 150 (62.0) | 3744 (63.3) |
| Birth weight in grams | Mean ± SD | 3195 ± 620 | 3151 ± 653 | 3131 ± 607 | 3148 ± 609 |

*Data are presented as numbers (%). RhD, Rhesus D; VD, Vaginal Delivery; PROM, Premature rupture of membranes; PIH, Pregnancy Induced Hypertension; APH, Antepartum* *Hemorrhage; PPH, Postpartum Hemorrhage*


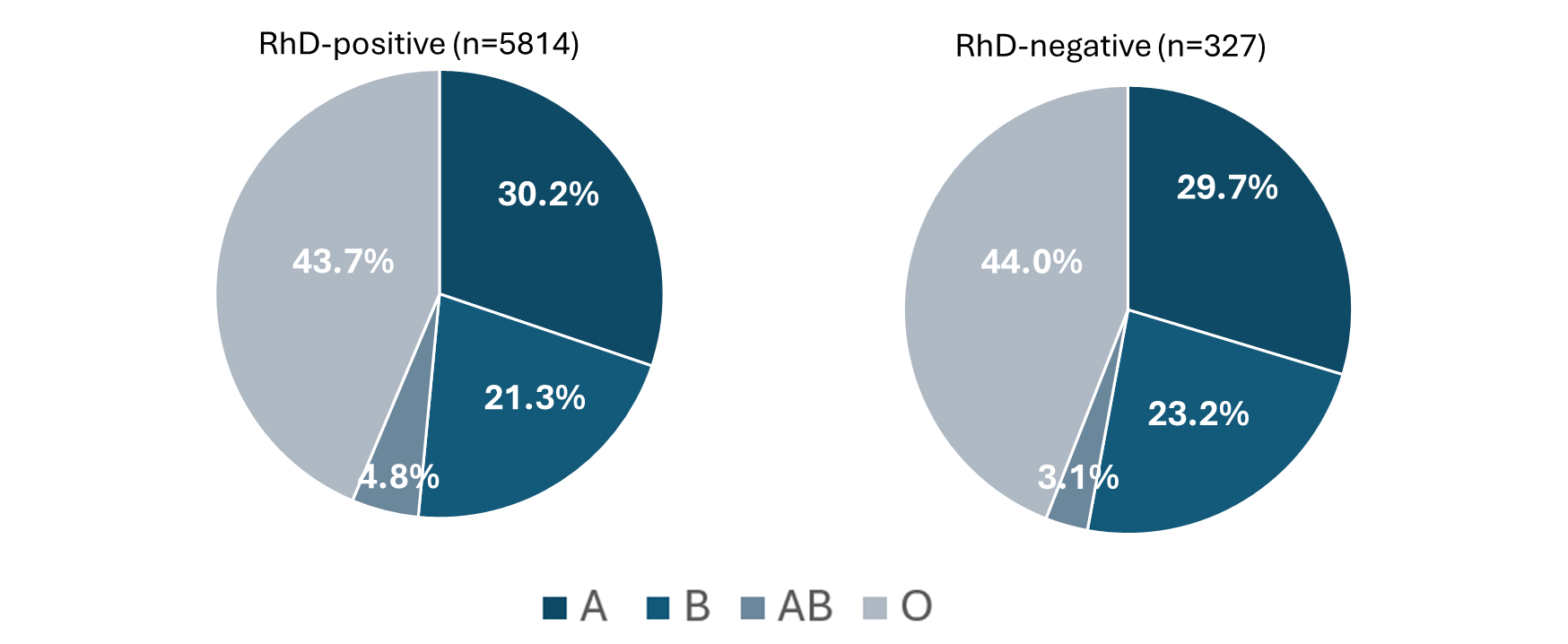


***Figure S2:*** *Distribution of maternal blood group and Rhesus D status among screened pregnancies in public hospitals in eastern Ethiopia, 2024 (n= 6141).*
